# Effect of microstructure change on permeability of flax-fiber reinforced silty clay soaked with zinc-ion solution

**DOI:** 10.1038/s41598-020-68332-4

**Published:** 2020-07-09

**Authors:** Qiang Ma, Jun-chen Xiang, Nian-ze Wu, Heng-lin Xiao

**Affiliations:** School of Civil Engineering and Environment, Hubei University of Technology, Wuhan, People’s Republic of China

**Keywords:** Environmental chemistry, Environmental impact

## Abstract

With the application of fiber-reinforcement technology, the mechanical properties of silty clay are improved with fiber reinforcement. However, the variation of permeability coefficient and other parameters of flax-fiber reinforced silty clay have not been sufficiently studied. In this study, the permeability of flax-fiber reinforced silty clay soaked with zinc-contaminated solution under different osmotic pressure was tested by a flexible-wall permeameter, and the effects of zinc-ion concentration and confining pressure on the permeability of flax-fiber reinforced silty clay were studied. Genius XRF was employed to detect the types and quantity of metal elements in the specimens, thereafter, the reasons for the change of permeability were explained from chemical and microscopic perspective. The results showed that the permeability coefficient of flax-fiber reinforced silty clay decreased significantly with the increase of zinc-ion concentration in a low concentration (about 1–10 mg L^−1^). While in a high concentration (about 100 mg L^−1^), the permeability coefficient of flax-fiber reinforced silty clay changed little with the increase of zinc-ion concentration. While the flax-fiber reinforced silty clay was not soaked with zinc-ion solution, the permeability coefficient of the specimen increased with the increase of confining pressure. However, when the flax-fiber reinforced silty clay was soaked with zinc-contaminated solution, the permeability coefficient first decreased and then tended to be constant with the increase of confining pressure. With the increase of confining pressure, the porosity of flax-fiber reinforced silty clay decreased, and with the increase of zinc-ion concentration, the porosity of flax-fiber reinforced silty clay first increased and then decreased.

## Introduction

In recent years, with the rapid development of industry, the pollution degree of industrial development area has been increasing^1–3^. There are some engineering projects built on the ground of demolished landfills, and residual contaminants in the subgrade always change the physical and chemical properties of the ground soil^4–6^. Depending on the survey bulletin of soil pollution in China published on April 17, 2018, the survey covers all of China's inland farmland, which included woodlands, grasslands, unused land and construction land, about 6.3 × 10^12^ m^2^. The result showed that the environmental in industrial wasteland was seriously polluted, and the soil pollution was common around the world. Among those pollutants, one class of the most serious pollutants is heavy metals. However, the eight kinds of heavy metals, including Cr, Ni, Cu, Zn, As, Cd, Hg and Pb, which accounted for the main part of inorganic pollution, and the average concentrations of them were 63.04, 26.18, 38.17, 137.72, 13.39, 0.68, 0.31 and 47.34 mg kg^−1^, respectively^7^. Among them, Cd, Hg and Pb are severe contamination, Cu and Zn are moderate contamination, and As, Cr and Ni are mild contamination^8–11^. It can also be seen that the average concentration of zinc is the highest, so the study on zinc pollution has become a hot topic. However, in the engineering, most polluted soil is regarded as natural soil. The neglected heavy metal ions affect the permeability of the soil, resulting in the overall settlement or uneven settlement of the foundation after rainfall, which brings a great potential trouble to the project^12,13^.

Fiber-reinforcement has a significant effect on mechanical and porous behavior of soil. Therefore, fiber-reinforcement technology has been widely concerned. Many scholars have done research on the mechanics and permeability of fiber reinforced soil. The influence of the physical structure of flax-fibers on soil mechanical properties was investigated by Van^14^. As shown in the results, both stretching and bending abilities of the flax-fiber reinforced soil was greater than the natural soil. The permeability of granular soils was checked through standard permeability test methods by Saghari and Bagheri^15^, and they evaluated the influence of polymer-fiber on soil permeability coefficients with different length of fibers. Ma^16^ carried out a series of laboratory triaxial tests to investigate the fiber reinforcement mechanism, and to study the characteristics of flax-fiber reinforced clay, thereafter, he found that the optimal reinforcement rate for clay was 0.8%. Tong^17^ put forward that a combined bamboo strips and flax-fiber reinforcement method of the clay. Through a series of triaxial compression tests, it was found that the addition of bamboo strips and flax-fiber improved the shear strength of the clay, and the deformation resistance of the clay was also improved. Those studies provided the experimental basis for investigating the mechanical properties of fiber reinforced soil, and it can be seen that mechanical properties are of great significance for practical engineering. However, in the engineering, it is not enough to only study the mechanical properties of clay, its permeability must be paid attention as well. So, it is essential to study the permeability of clay to improve the stability of engineering and reduce safety risks.

The permeability of heavy metal contaminated soil has been a hot topic in geotechnical engineering. As stated in the literatures, both porosity and permeability coefficient are essential indicators that reflect the permeability of soil^18–20^. The existence of heavy metal causes the change of soil internal structure and weakens the cementation between soil particles^21–24^. Therefore, it is important to effectively analyze the permeability of heavy metal contaminated soil. Turer^25^ investigated the related performance of kaolin polluted by Pb(NO_3_)_2_ and Zn(NO_3_)_2_ solutions, as shown in the results, while the concentration of Pb^2+^ and Zn^2+^ increased, the expansion rate and permeability of kaolin increased. Further, heavy metal ions increased the maximum dry density and permeability coefficient of silty clay, and reduced the optimum moisture content and liquid limit of silty clay. This result was shown by Shariatmadari^26^. Zhang^27^ carried out hydraulic conductivity experiments by the flexible-wall permeameter, in the results, when the CuCl_2_ concentration was less than 0.5 g L^−1^, the void ratio and the hydraulic conductivity of soil slightly decreased with the increase of solution concentration. Once the CuCl_2_ concentration increased over 0.5 g L^−1^, with the increase of osmotic concentration, both the void ratio and hydraulic conductivity values increased.

Overall, there are many researches on the permeability of heavy metal polluted silty clay, whereas few researches on the permeability of fiber reinforced silty clay polluted by heavy metal. Only investigating the permeability of fiber-reinforced clay and polluted silty clay cannot meet the needs of actual engineering. In the case of certain circumstances, such as the effect of polluted rainwater on the permeability of the reinforced foundation and the influence of polluted water on the permeability of the reinforced embankment, and it is not possible to solve those problem by studying the existing references, so it is necessary to investigate the permeability of fiber-reinforced silty clay polluted by heavy metal. Therefore, in order to provide a certain solution to the above engineering problems, the permeability coefficient of the flax-fiber reinforced silty clay soaked with zinc-contaminated solution was investigated in this study. The effects of different zinc-ion concentrations and different confining pressure on the permeability of flax-fiber reinforced silty clay were discussed, and the causes of the test results were analyzed from chemical and microscopic perspective.

## Materials and methods

### Selection of soil specimen

**Figure 1:**
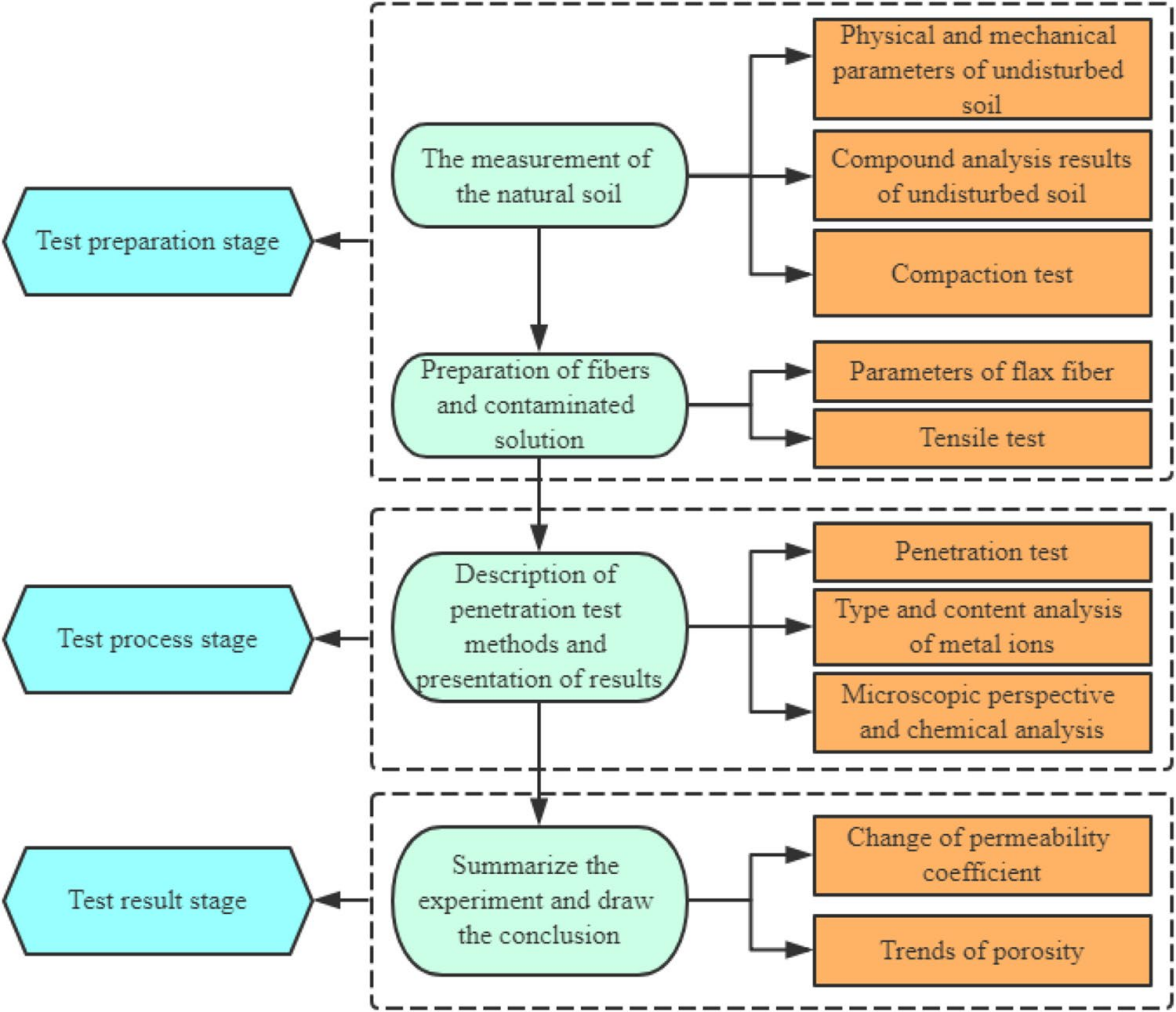
The mind map of this experiment.

The soil specimens used in this study were taken from the foundation pit beside the civil building of Hubei University of Technology in Wuhan. The depth of the moved soil was about 2 m, and the soil was silty clay. The mind map of this experiment is shown in Fig. 1.

The physical and mechanical properties parameters of the undisturbed soil are shown in Table 1, and the compound analysis results are listed in Table 2.

**Table 1:** Physical and mechanical parameters of undisturbed soil.

Parameters	Numerical value
Density (g cm^−3^)	1.82
Specific gravity	2.69
Specific gravity	32.00
Plastic limit (%)	48.20
Liquid limit (%)	20.10

**Table 2:** Compound analysis results of undisturbed soil.

Compound species	Content (%)
SiO_2_	63.17
MgO	1.00
CaO	0.43
Al_2_O_3_	15.95
Fe_2_O_3_	6.85
K_2_O	2.02
Na_2_O	0.37
TiO_2_	0.91
P_2_O_5_	0.05
MnO	0.06
Ignition lost	9.20

**Figure 2:**
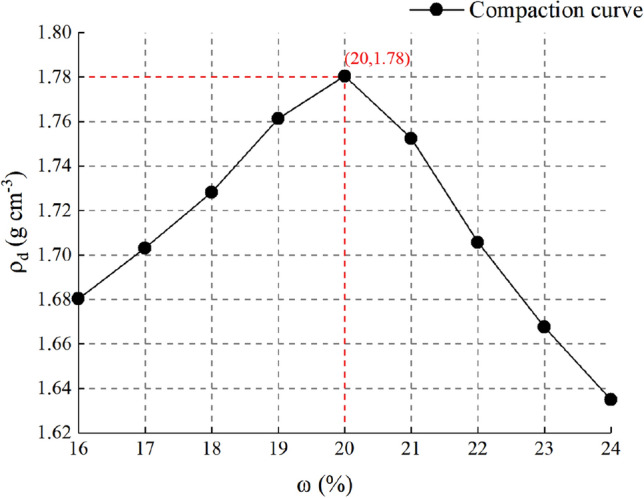
Maximum dry density-optimum moisture content curve of the silty clay.

Moisture content has a significant impact on mechanical properties and permeability of silty clay^28,29^. Therefore, the optimum moisture content and maximum dry density of the silty clay were detected by light compaction test^30^. The undisturbed soil is crushed with a compaction hammer, and soil particles smaller than 5 mm were screened through a sieve basin^30^. The prepared specimen was put into the light compactor, and the specimen was compacted in three layers, each layer was compacted for fifteen times. The mould of compaction test is shown in Supplementary Fig. S1(a) online. The result of light compaction test is shown in Fig. 2. The optimal moisture content of undisturbed soil was 20%(*ω*), and the corresponding maximum dry density was 1.78 g cm^−3^(*ρ*)^13^.

In the permeability test, the size of the specimen preparation mold is 70 mm(*d*) × 20 mm(*h*), as shown in Supplementary Fig. S1(b) online. So the mass(*m*) of silty clay is 136.9 g, and the calculation formula is shown in Eq. 1.

1 $$m = \rho \times \left( {\frac{\pi }{4} \times d^{2} \times h} \right)$$

where *m* is the mass of specimen in the permeability test, *ρ* is the maximum dry density of the specimen, *d* is the diameter of the specimen, *h* is the height of the specimen.

### Selection of fibers

The fiber used in this study was flax-fiber, had long and thin appearance and rough surface. The parameters of flax-fiber are shown in Table 3.

**Table 3:** Parameters of flax fiber.

Parameters	Numerical value
Length	20 mm
Diameter	0.35 mm
Ultimate tensile strength	127.54 MPa
Tensile modulus	0.51 GPa
Cross-section shape	Roughly circular

**Figure 3:**
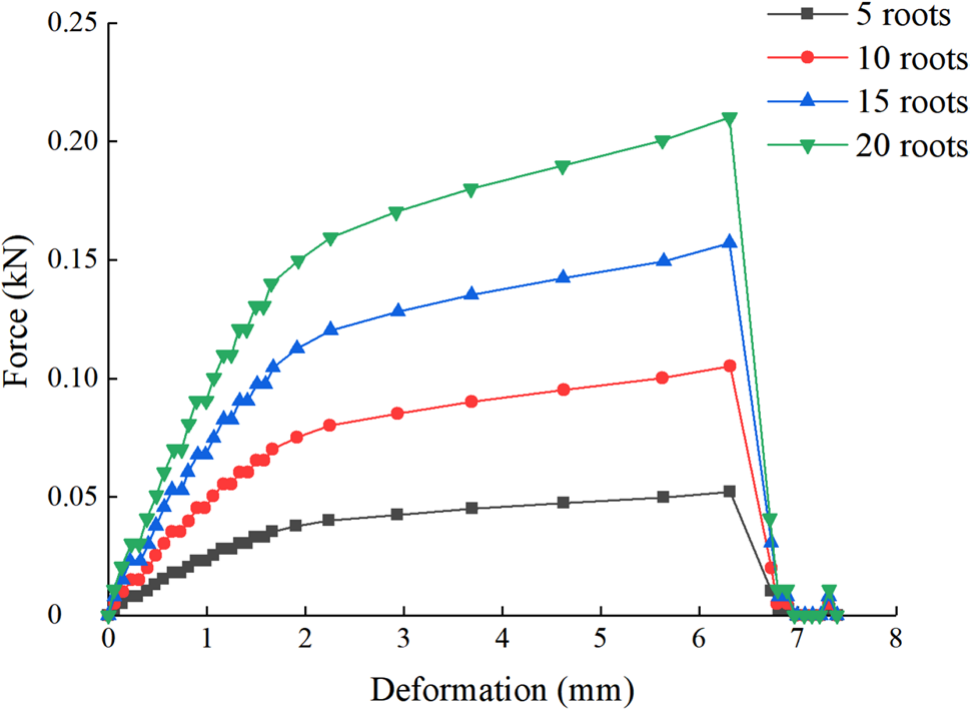
Tension-deformation curve of flax fiber.

The axial tension-deformation curve of the flax-fiber was obtained by a series of tensile tests. Four groups of tensile tests were conducted with 5, 10, 15 and 20 flax fibers, respectively. The test results are shown in Fig. 3.

**Figure 4:**
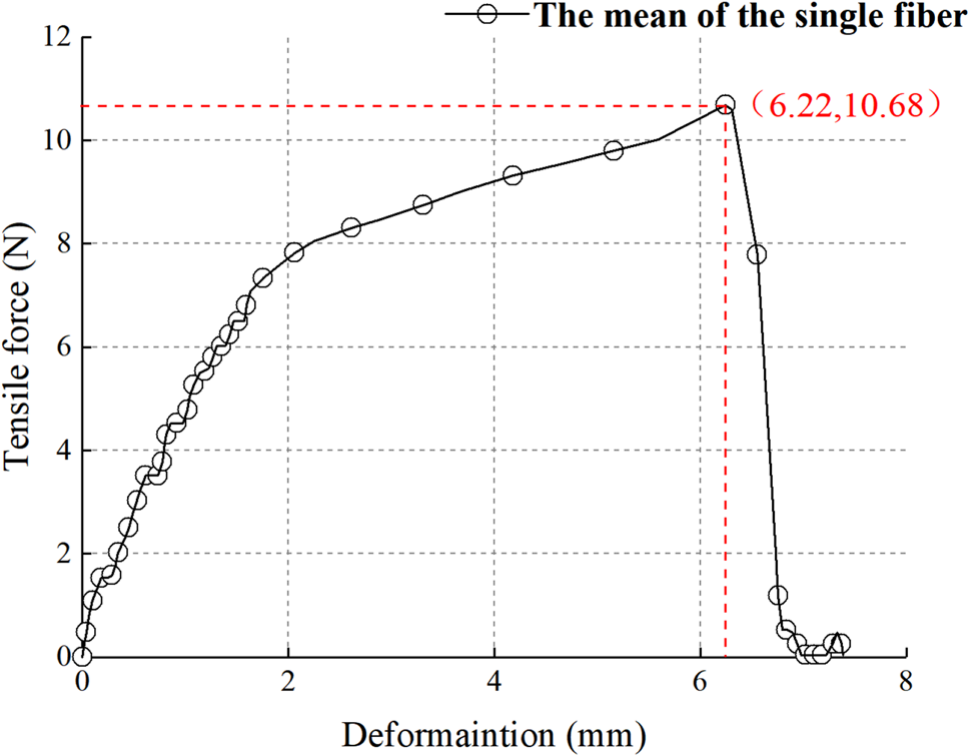
Tensile force vs. elongation curve of flax fiber.

In order to reduce the test error, the average values of the four groups of the test results were regarded as the test results, and the average results are shown in Fig. 4.

According to reference^16^, the optimum fiber content of silty clay was 0.8%(*ω*_*m*_). Therefore, the mass percent of flax-fiber in this study is 0.8%(*ω*_*m*_). The mass(*m*_*f*_) of the fiber is 1.1 g, and the calculation formula is shown in Eq. 2.

2 $$m_{f} = m \times \omega_{m}$$

where *m*_*f*_ is the mass of flax-fiber in the specimen, *m* is the mass of the specimen, which is consistent with Eq. 1, *ω*_*m*_ is the optimal mass fraction of flax-fiber in the specimen.

### Preparation of polluted silty clay

In this study, Zn(NO_3_)_2_ was selected as the solute. The reason was that the average concentration of zinc is the highest among heavy metals and the contamination of zinc is the most extensive^7^. Therefore, Zn^2+^ was selected as the cation. For the anion, SO_4_^2-^ and Cl^-^ were destructive to the silty clay in a certain extent. Compared with those anions, NO_3_^-^ had a least destructive effect on the silty clay. The solubility of Zn(NO_3_)_2_ is high, and Zn(NO_3_)_2_ solid dissolved quickly in water to form a colorless and transparent Zn(NO_3_)_2_ solutions. The chemical equation is shown as Eq. 3:

3 $${\text{Zn}}({\text{NO}}_{3} )_{2} \, ({\text{s}}) \to {\text{Zn}}^{2 + } + ({\text{aq}}) + 2{\text{NO}}_{3}^{ - } \, ({\text{aq}})$$

where s and aq are the states of matter, where s is solid, aq is solution.

If the pH of the solution is around 7, the zinc-ions will hydrolyze to produce Zn(OH)_2_ precipitation, and the chemical equation is shown as Eq. 4:

4 $${\text{Zn}}^{2 + } \,({\text{aq}}) + 2{\text{OH}}^{ - } \,({\text{aq}}) \to {\text{Zn}}({\text{OH}})_{2} \, ({\text{s}})$$

where s and aq are the states of matter, where s is solid, aq is solution, which is consistent with Eq. 3.

In order to eliminate the production of Zn(OH)_2_ precipitation and interference of other irrelevant ions, the solution selected in this experiment was distilled water. The concentrations of zinc-ions in the solution were determined as 0 mg L^−1^, 1 mg L^−1^, 10 mg L^−1^ and 100 mg L^−1^, respectively.

The undisturbed soil was placed in the oven to dry for 1 day, and then was filtered through a sieve basin with the diameter of 5 mm for later use. The silty clay with 136.9 g and the flax-fiber with 1.1 g were weighed by an electronic scale, and then were put into a mixing bowl. The prepared Zn(NO_3_)_2_ solution with the concentration of 0 mg L^−1^, 1 mg L^−1^, 10 mg L^−1^ and 100 mg L^−1^ was sprayed evenly into the mixing bowl, and the specimens were mixed by artificial mixing. Then, the specimens were made by compaction. Each specimen was equally divided into two layers to be compacted, and the falling height of the compaction hammer was 15 mm (as shown in Supplementary Fig. S2(a) online). After preparation, the specimens were sealed with plastic film (as shown in Supplementary Fig. S2(b) online) and placed in an incubator at 20 °C for 24 h.

### Test methods

Permeability test: The variable parameters of this test are shown in Table 4. As can be seen from Table 4, the influencing factors in this test are zinc-ion concentration, confining pressure and osmotic pressure difference. Therefore, the total number of specimens required is 64.

**Table 4:** Parameters of each variable in permeability test.

Parameters	Numerical			
Zinc-ion concentration (mg L^−1^)	0	1	10	100
Confining pressure (kPa)	10	20	30	40
Osmotic pressure difference (kPa)	3	6	9	12

The preserved specimens were taken out and put into the flexible-wall permeameter for permeability test. The test instrument is shown in Supplementary Fig. S3(a) online.

The specimen was put into the specimen chamber (as shown in Supplementary Fig. S3(b) and Fig. S3(c) online). The specimen was saturated at 35 kPa confining pressure and 15 kPa osmotic pressure difference (upper pressure minus lower pressure), and the saturation time was 48 h. After saturation, the appropriate confining pressure and osmotic pressure difference were set according to Table 4. Then, the permeability test could be started, and the permeability time of each specimen was 6 h.

Consolidation test: The zinc-ion concentration, consolidation pressure and consolidation time of the test are shown in Table 5, and the total number of specimens is 16.

**Table 5:** Parameters of each variable in consolidation test.

Parameters	Numerical			
Zinc-ion concentration (mg L^−1^)	0	1	10	100
Consolidation pressure (kPa)	10	20	30	40
Consolidation time (h)	24	24	24	24

The consolidation test apparatus is the Model of GZQ-1 Full Automatic Pneumatic Consolidation Test Apparatus. The specimens with different zinc-ion concentrations were placed in the consolidation apparatus respectively, as shown in Supplementary Fig. S4 online. The specimen was placed in the chamber and the consolidation rate was set to 0.01 mm h^−1^. The test of time and consolidation pressure were set consistent with Table 5.

## Results and discussion

### Permeability coefficients of specimens under different confining pressures

**Figure 5:**
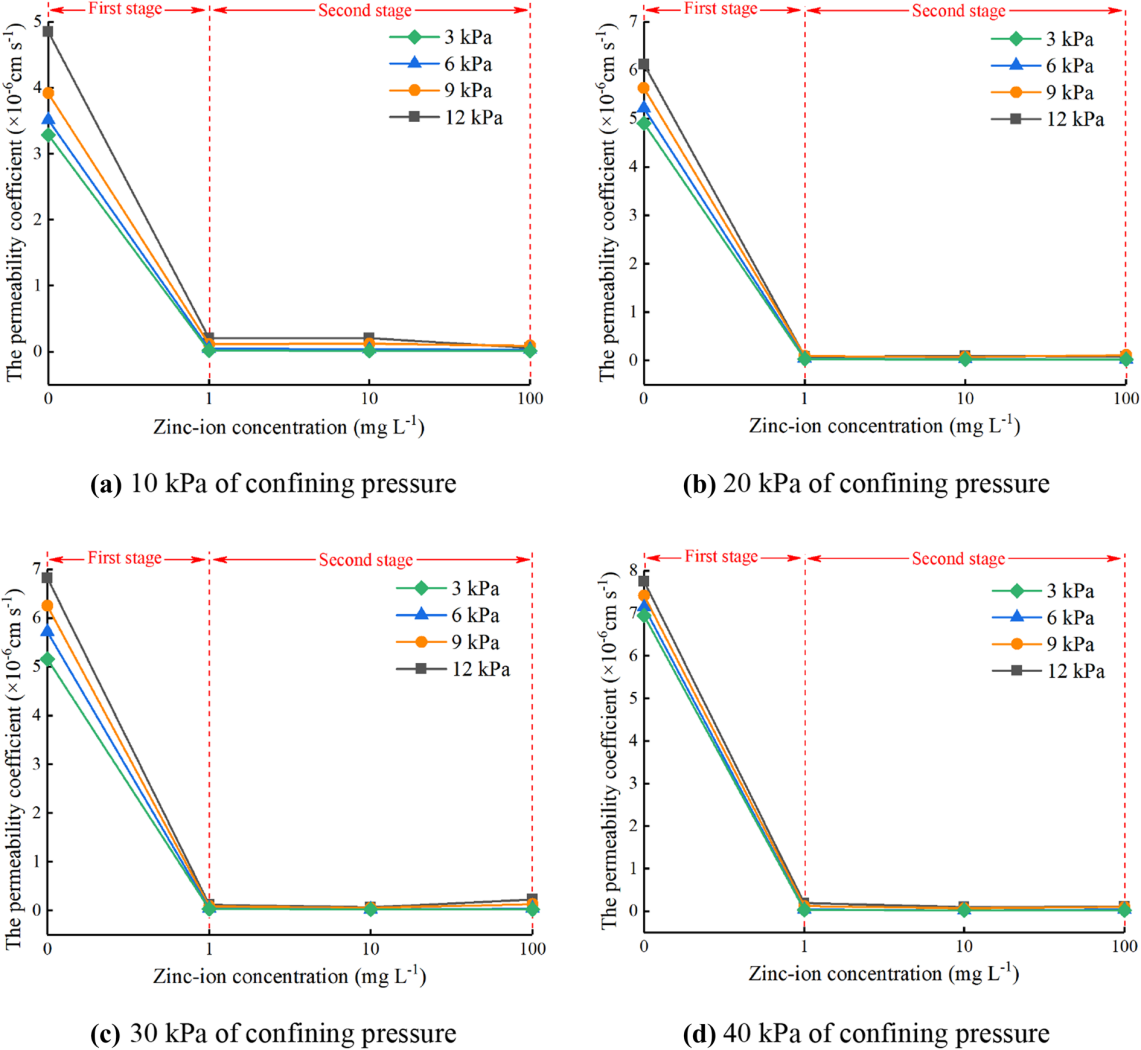
Permeability coefficient of specimens under different confining pressures. (**a**) 10 kPa of confining pressure, (**b**) 20 kPa of confining pressure, (**c**) 30 kPa of confining pressure, (**d**) 40 kPa of confining pressure.

The four confining pressures selected in this test are 10 kPa, 20 kPa, 30 kPa and 40 kPa. Due to the confining pressures encountered in actual engineering are generally 0–40 kPa, so the confining pressures selected in this test meet the engineering needs. The permeability coefficients of specimens under different confining pressures are shown in Fig. 5.

As can be seen from Fig. 5, the overall trend of the permeability coefficient at four confining pressure is almost the same. The trend can be divided into two stages. The first stage is the rapid declining stage, which ranges from 0 to 1 mg L^−1^. With the increase of zinc-ion concentration, the permeability coefficient of the specimen drops obviously, and the slope of the curve is large. The second stage is the stable stage, ranging from 1 to 100 mg L^−1^. In this stage, with the increase of zinc-ion concentration, the permeability coefficient of the specimen changes little, and the curve tends to be stable. While the zinc-ion concentration is 0 mg L^−1^, the permeability coefficient of the specimen is greater than that of other concentrations. Compared with unpolluted silty clay, the permeability coefficient of polluted silty clay decreased greatly, about from 6×10^−6^ to 0.2×10^−6^ cm s^−1^. With the increase of osmotic pressure difference, the permeability coefficient of the specimen shows an increasing trend. The rate of increase is large while the zinc-ion concentration is 0 mg L^−1^. On the contrary, while the concentration is 1 mg L^−1^, 10 mg L^−1^ and 100 mg L^−1^, the rate of increase is small. It can be seen that the permeability of silty clay soaked with zinc-contaminated solution is obviously different from that of the original clay. However, with the increase of zinc-ion concentration, the permeability of the polluted silty clay has no obvious changes.

### Permeability coefficients of specimens with different zinc-ion concentrations

**Figure 6:**
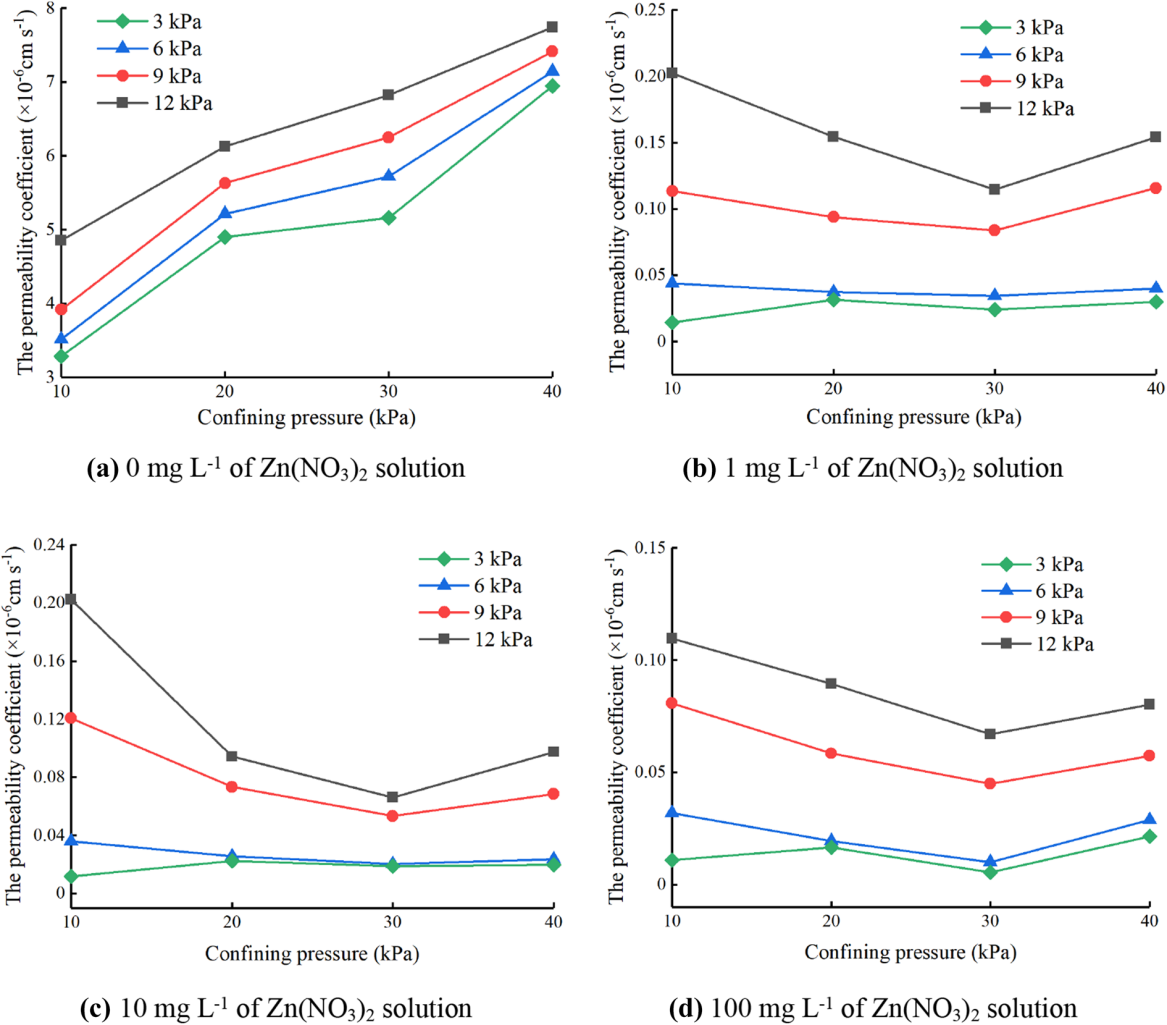
Permeability coefficients of specimens under different zinc-ion concentration solution. (**a**) 0 mg L^−1^ of Zn(NO_3_)_2_ solution, (**b**) 1 mg L^−1^ of Zn(NO_3_)_2_ solution, (**c**) 10 mg L^−1^ of Zn(NO_3_)_2_ solution, (**d**) 100 mg L^−1^ of Zn(NO_3_)_2_ solution.

Permeability test was conducted at the zinc-ion concentration of 0 mg L^−1^, 1 mg L^−1^, 10 mg L^−1^ and 100 mg L^−1^, respectively. The permeability coefficients of specimens under different zinc-ion concentration solutions are shown in Fig. 6.

As can be seen from Fig. 6, when the zinc-ion concentration is 0 mg L^−1^, the permeability coefficient increases with the increase of confining pressure. While the confining pressure is 10–30 kPa, the uptrend of the curve is slow, but when the confining pressure is 30–40 kPa, the slope of the curve becomes larger, and the uptrend becomes faster. However, while the zinc-ion concentration is 1 mg L^−1^, 10 mg L^−1^ and 100 mg L^−1^, the permeability coefficient first decreased and then increased with the increase of confining pressure. Under the same confining pressure, the greater the osmotic pressure difference, the greater the permeability coefficient of the specimen.

### Porosity of specimens with different zinc-ion concentrations

**Figure 7:**
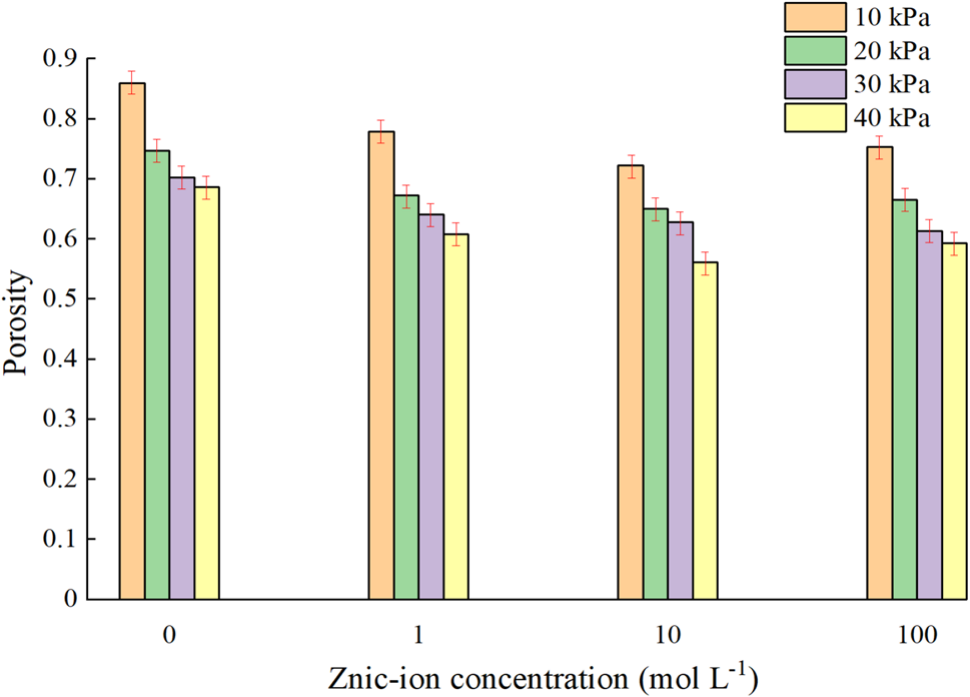
Porosity of specimens under different concentration of zinc-ion solutions.

At 10, 20, 30 and 40 kPa confining pressures, the porosity of the flax-fiber reinforced silty clay soaked with four different zinc-ion concentrations solutions is shown in Fig. 7. (Explanation: Each bar has a red line of symbol, the upper end of them indicates the maximum value, and the lower end indicates the minimum value.)

As can be seen from Fig. 7, while the flax-fiber reinforced silty clay is not soaked with zinc-contaminated solution, the porosity of the specimen ranges from 0.7 to 0.9, and the maximum porosity is approximately 0.87. However, while the specimen is soaked with zinc-contaminated solution, the porosity of the specimen decreases obviously and maintains in the range of 0.5–0.7, and the maximum porosity of the specimen is about 0.68. At the same zinc-ion concentration, with the increase of confining pressure, the porosity of flax-fiber reinforced silty clay decreases. When the zinc-ion concentration is low (about 0–1 mg L^−1^) and under the same confining pressure, the porosity of the flax-fiber reinforced silty clay decreases with the increase of the zinc-ion concentration, and the rate of decrease is large. When the zinc-ion concentration is high (about 10–100 mg L^−1^) and under the same confining pressure, the porosity of the flax-fiber reinforced silty clay increases with the increase of the zinc-ion concentration, but the rate of decrease is small. Therefore, it can be seen that in the low-concentration environment, the change of zinc-ion concentration has a great influence on the specimen porosity, once in the high-concentration environment, the change of zinc-ion concentration has little effect on the specimen porosity. While the concentration is about 10 mg L^−1^, the minimum porosity of the specimen is 0.58. It can be inferred that the zinc-ion concentration which has the greatest influence on the porosity of the specimen is about 10 mg L^−1^.

### Genius XRF analysis

**Figure 8:**
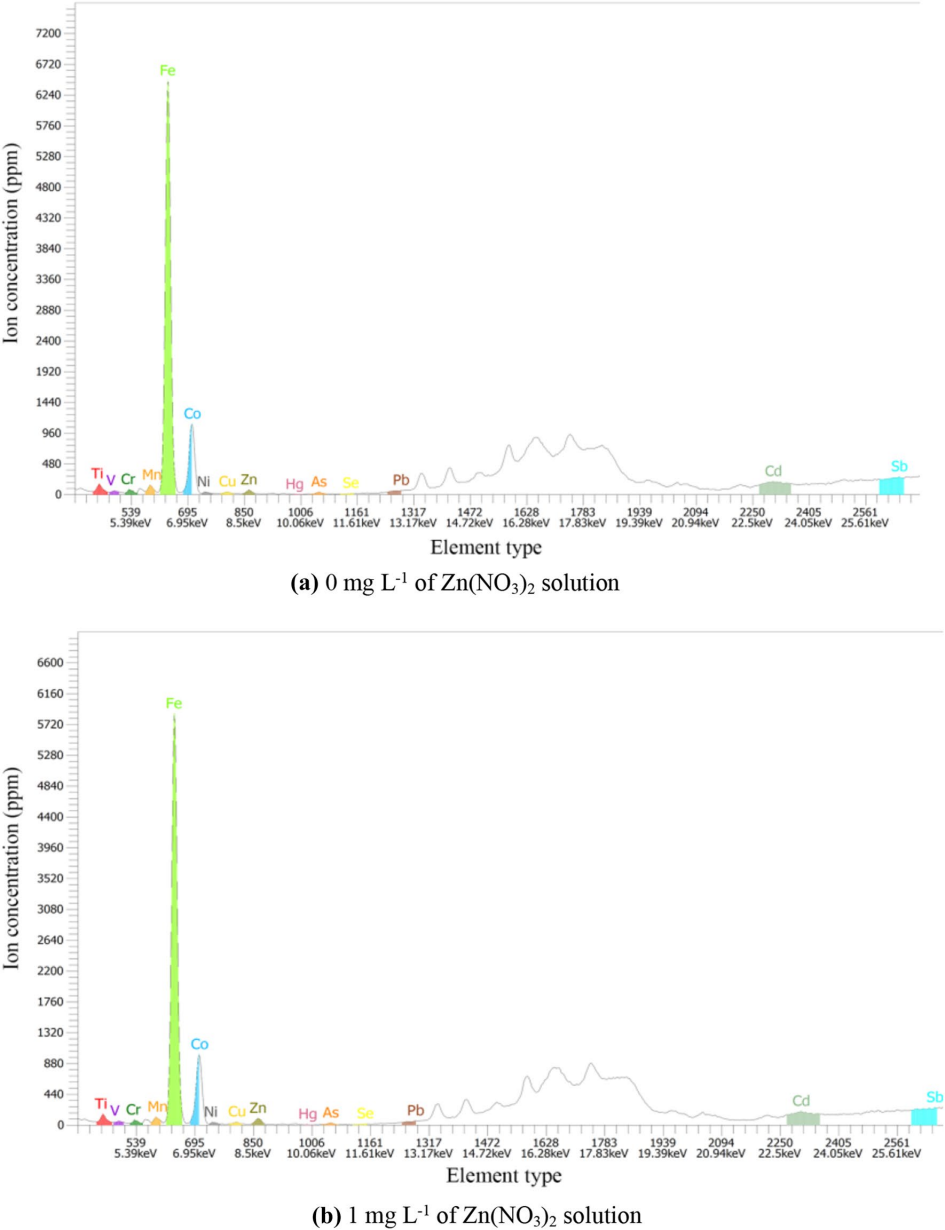

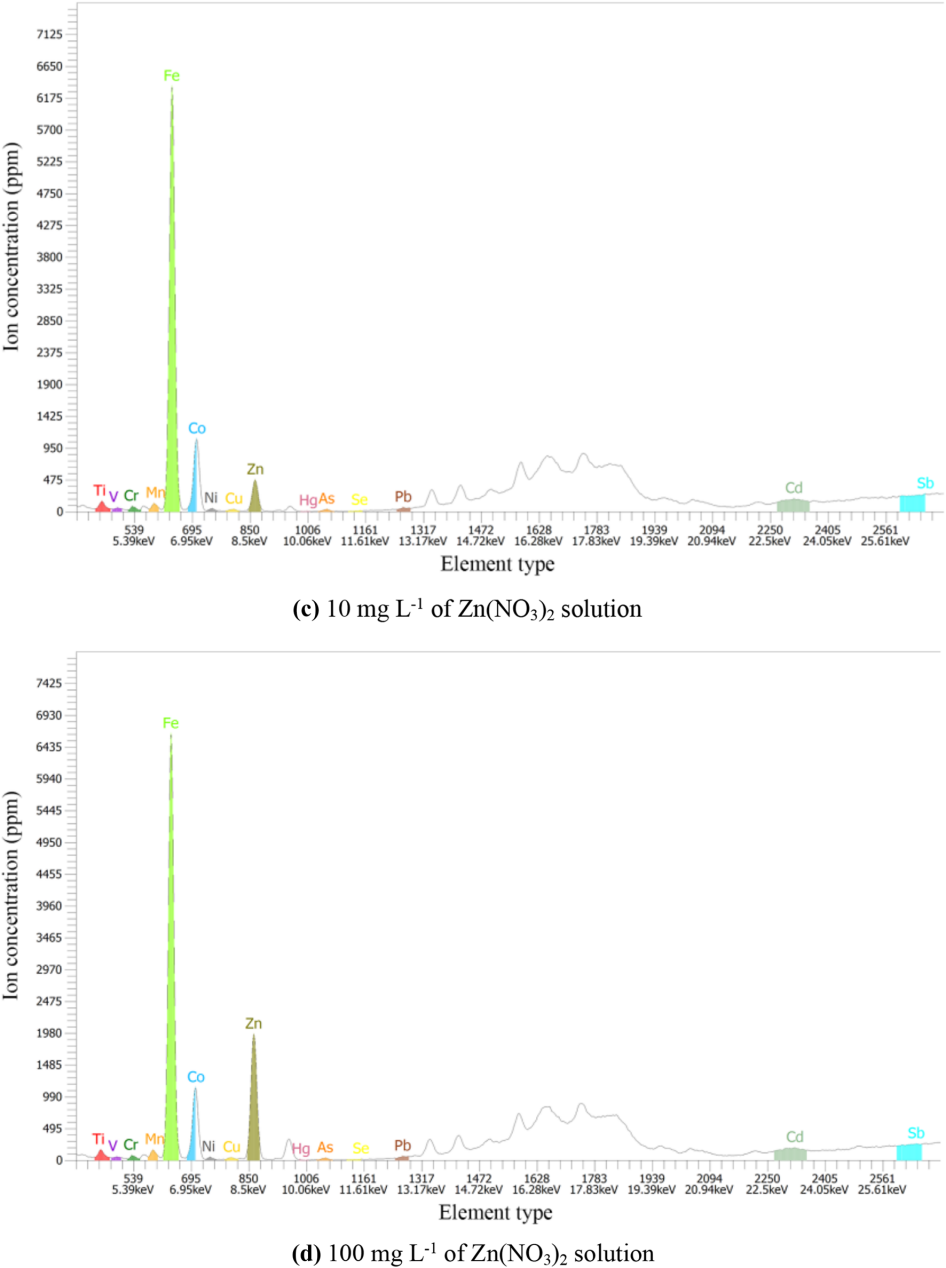
Metallic element analysis results of different zinc-ion concentrations. (**a**) 0 mg L^−1^ of Zn(NO_3_)_2_ solution, (**b**) 1 mg L^−1^ of Zn(NO_3_)_2_ solution, (**c**) 10 mg L^−1^ of Zn(NO_3_)_2_ solution, (d) 100 mg L^−1^ of Zn(NO_3_)_2_ solution.

In order to prove that the only metal factor affecting the permeability of the specimen is Zn, so Genius XRF was used to test the specimens with different concentrations of zinc-ions under natural conditions. The test results are shown in Fig. 8.

As can be seen from Fig. 8, the metals with high content in the four specimens are Fe, Zn, Co, Sb, Cd, etc. With the increase of zinc-ion concentration in the solution, the content of zinc-ion in the flax-fiber reinforced silty clay increases obviously. In Fig. 8a, the concentration of zinc is 96 ppm, and the concentration of zinc increases to 1980 ppm in Fig. 8d. But the types and quantity of other metallic ions in the specimen have a little change. It can be seen that the concentration of zinc-ions in the solution does not affect the types and quantity of other metallic ions in the specimen. From a single variable perspective, the influence of other metal ions except the zinc-ion can be excluded in this study. Thus, it is proved that the zinc-ion is the only ion that has influence on the permeability of flax-fiber reinforced silty clay.

### Microanalysis results and chemical explanation of specimens

**Figure 9:**
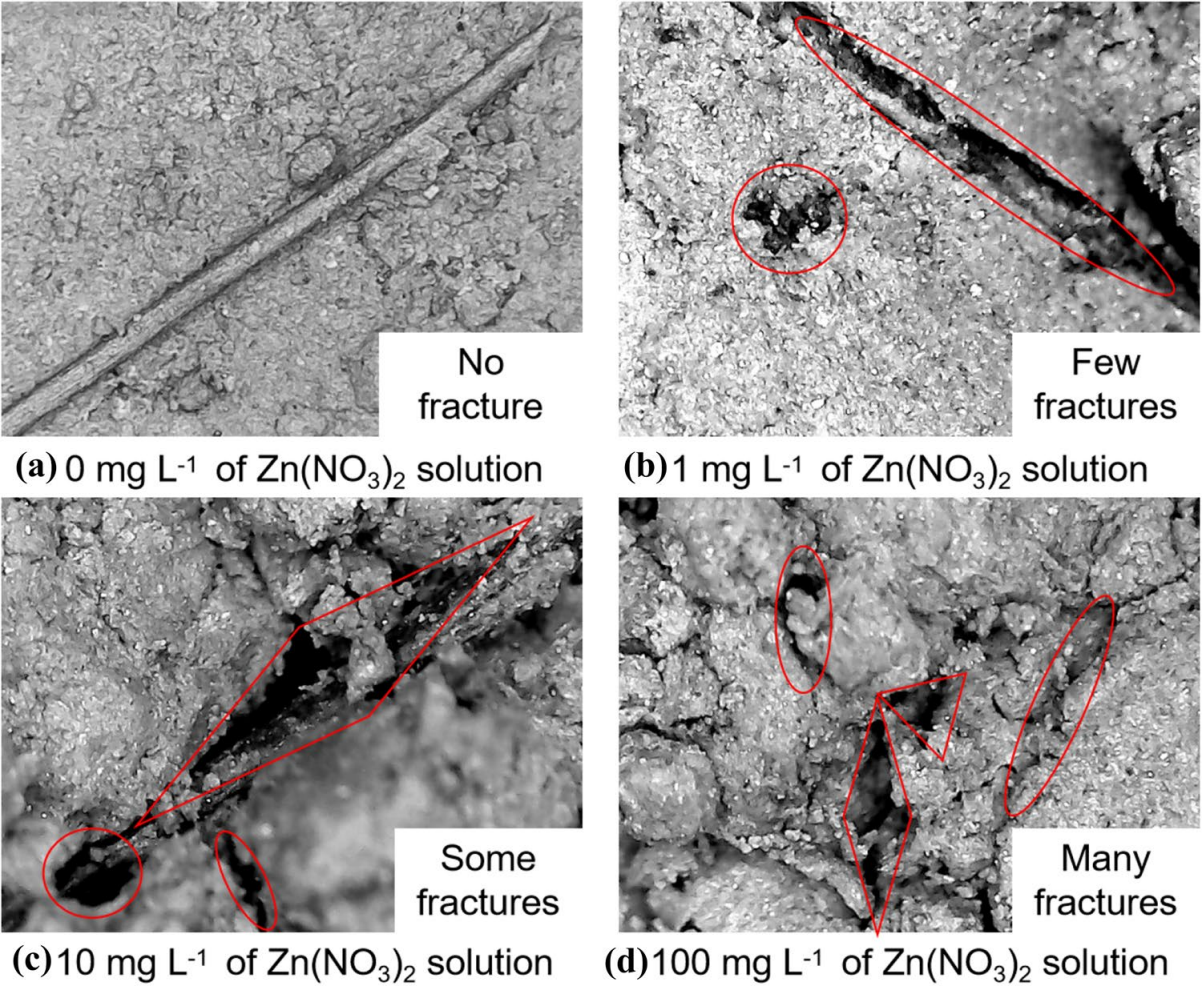
Micrograph of specimens with different zinc-ion concentrations (Magnification ratio: 1:100). (**a**) 0 mg L^−1^ of Zn(NO_3_)_2_ solution, (**b**) 1 mg L^−1^ of Zn(NO_3_)_2_ solution, (**c**) 10 mg L^−1^ of Zn(NO_3_)_2_ solution, (**d**) 100 mg L^−1^ of Zn(NO_3_)_2_ solution.

Under natural conditions, the specimens with four zinc-ion concentration solutions were photographed by the microscope after permeability test. The micrograph scale is 1:100, as shown in Fig. 9.

As can be seen from Fig. 9, with the increase of zinc-ion concentration, the number and size of fractures between soil particles first increase and then tend to be stable. While the flax-fiber reinforced silty clay is not soaked with zinc-contaminated solution (as shown in Fig. 9a), there are almost no fractures in the silty clay. However, once the flax-fiber reinforced silty clay is soaked with zinc-contaminated solution, the fractures begin to appear. At a low concentration (about 1–10 mg L^−1^, as can be seen from Fig. 9a to b), the fractures gradually become greater with the increase of zinc-ion concentration. But in a high concentrations (about 100 mg L^−1^, as shown in Fig. 9d), the fractures change little with the increase of zinc-ion concentration. The reason for this phenomenon is that at the stage of about 1–10 mg L^−1^, the chemical reaction of zinc-ions with the hydroxide-ion in the silty clay is due to the increase of zinc-ions concentration, and the Zn(OH)_2_ precipitates adhere to the surface of the soil particles (Supported in Section “XRD analysis before and after permeability test”). Moreover, the mineral compositions in silty clay have adsorption and complexation with zinc-ions, which block the effective seepage aperture and reduce the permeability coefficient^31^. It indicates that the zinc-ions play a dominant role in the silty clay structure at a low concentration. However, while the zinc-ion concentration is about 100 mg L^−1^, the quantity of mineral composition and hydroxide-ions in each specimen is approximately the same. So with the increase of zinc-ion concentration, the quantity of formed Zn(OH)_2_ precipitates is almost the same as that at the low concentration, and the structural damage degree of flax-fiber reinforced silty clay is almost unchanged^32^. Therefore, it can be seen that, at the high concentration, the permeability coefficient of flax-fiber reinforced silty clay has little change with the increase of zinc-ion concentration.

### XRD analysis before and after permeability test

**Figure 10:**
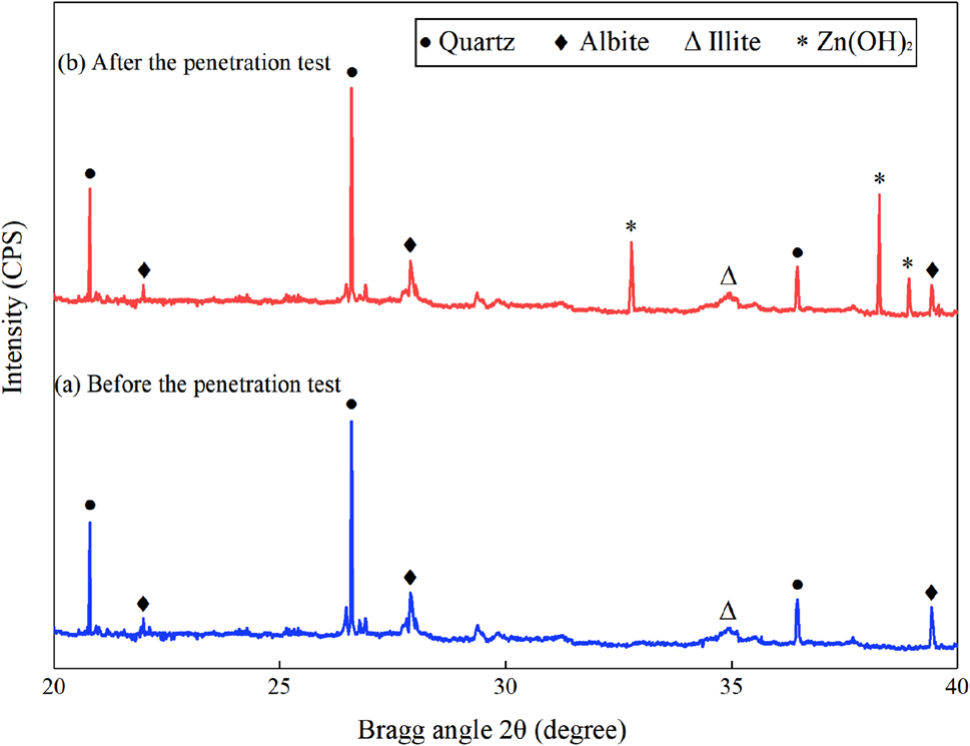
XRD of the specimen with 10 mg L^−1^ zinc-ion concentration. (**a**) Before the penetration test, (**b**) After the penetration test.

In order to further analyze the influence of zinc-ion on the specimens, XRD analysis was conducted on them before and after the permeability test. The XRD results are shown in Fig. 10.

As can be seen from Fig. 10, the content of many substances, such as quartz and albite, barely changed before and after the permeability test. The zinc hydroxide does not appear in the specimen before the test, as shown in Fig. 10a. However, it can be seen from Fig. 10b that the zinc hydroxide components appears in the specimen after hydrogen, and three peaks appears in 2θ = 32.78°, 38.27° and 38.96° (2θ is the diffraction angle, which refers to the angle between the X-ray and the diffraction line. θ is the diffraction half angle, which refers to the angle between the X-ray and the crystal plane), respectively. The results effectively support the reason explained in Section “Microanalysis results and chemical explanation of specimens”, and its regularity is consistent with the discussion by Mi^33^.

## Conclusion

In this study, relevant tests were conducted on the influence of different zinc-ion concentrations on the permeability coefficient of flax-fiber reinforced silty clay, and the main conclusions were as follows:

1. In a low concentration (about 1–10 mg L^−1^), the permeability coefficient of polluted flax-fiber reinforced silty clay decreases significantly with the increase of zinc-ion concentration. On the contrary, in the high concentration (about 100 mg L^−1^), the permeability coefficient of polluted flax-fiber reinforced silty clay is almost unchanged with the increase of zinc-ion concentration.
2. When the flax-fiber reinforced silty clay is not polluted, the permeability coefficient of the specimen increases with the increase of confining pressure. But when the zinc-ion concentrations are 1 mg L^−1^, 10 mg L^−1^ and 100 mg L^−1^, the permeability coefficient decreases first and then tends to be stable with the increase of confining pressure.
3. With the increase of confining pressure, the porosity of flax-fiber reinforced silty clay decreases. And with the increase of zinc-ion concentration, the porosity of flax-fiber reinforced silty clay increases first and then decreases.
4. The concentration of zinc-ion has an effect on the internal structure of silty clay. As observed under the microscope, the number and size of fractures increase with the increase of zinc-ion concentration.

## Supplementary information

Supplementary file1 (DOCX 1664 kb)
