# Supplementary material for: Effect of microstructure change on permeability of flax-fiber reinforced silty clay soaked with zinc-ion solution
Source: Sci Rep. 2020 Jul 9;10:11296. doi: 10.1038/s41598-020-68332-4 (PMC7347911; doi:10.1038/s41598-020-68332-4)
Supplement: Supplementary file 1 — Supplementary file1 (DOCX 1664 kb) [file 41598_2020_68332_MOESM1_ESM.docx]

**Effect of microstructure change on permeability of flax-fiber reinforced silty clay soaked with zinc-ion solution**

Qiang Ma, Jun-chen Xiang, Nian-ze Wu, Heng-lin Xiao*

School of Civil Engineering and Environment, Hubei University of Technology, Wuhan, People’s Republic of China

*Corresponding Author：E-mail: xiao-henglin@163.com，Tel: +86-189-7167-0600

**Supporting information captions**

**Supplementary Figure S1.** Compaction mould. (a) Compaction mould of compaction test, (b) Compaction mould of penetration test. （Those images was taken by J.C. on July 1, 2019 )

**Supplementary Figure S2.** Specimens preparation instructions. (a) Specimen preparation, (b) Finished specimens. （Those images were taken by J.C. on July 9, 2019)

**Supplementary Figure S3.** Test instrument description. (a) Flexible-wall permeameter, (b) Loading specimen, (c) Specimen chamber. （Those images were taken by J.C. on July 17, 2019)

**Supplementary Figure S4.** Description of consolidation apparatus. （The image was taken by J.C. on August 4, 2019)

| 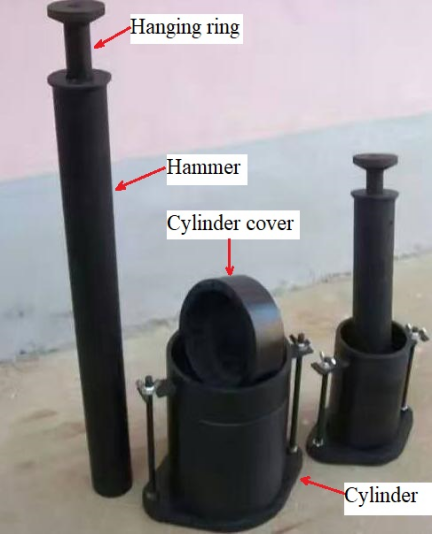 | 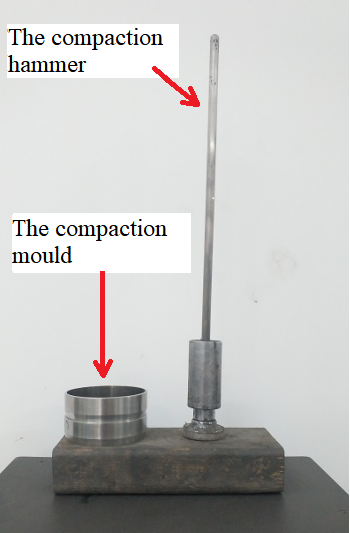 |
| --- | --- |
| (a) Compaction mould of compaction test | (b) Compaction mould of penetration test |

**Supplementary Figure S1.** Compaction mould

| 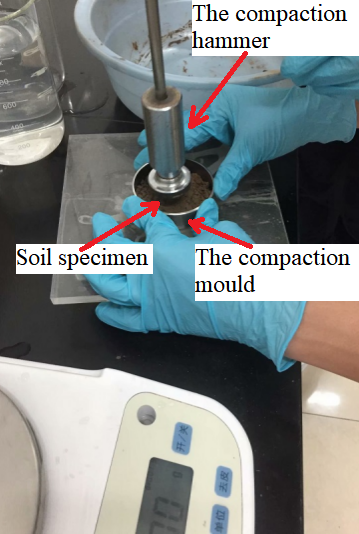 | 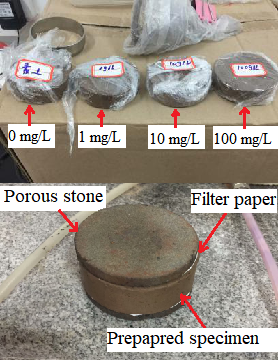 |
| --- | --- |
| (a) Specimen preparation | (b) Finished specimens |

**Supplementary Figure S2.** Specimens preparation instructions

| 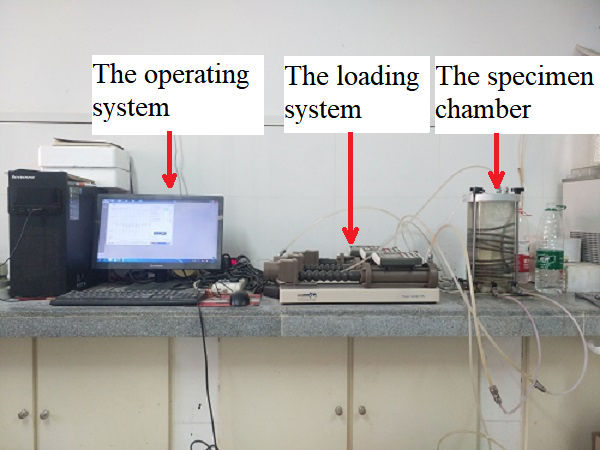 | |
| --- | --- |
| (a) Flexible-wall permeameter | |
| 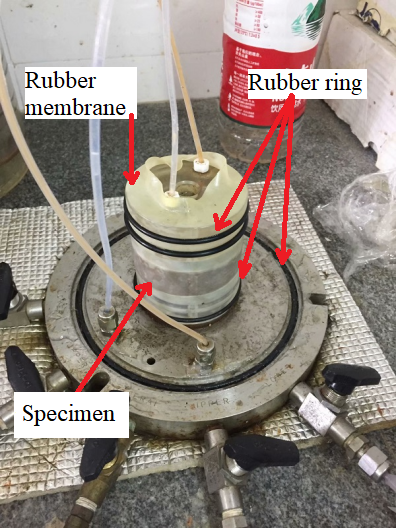 | 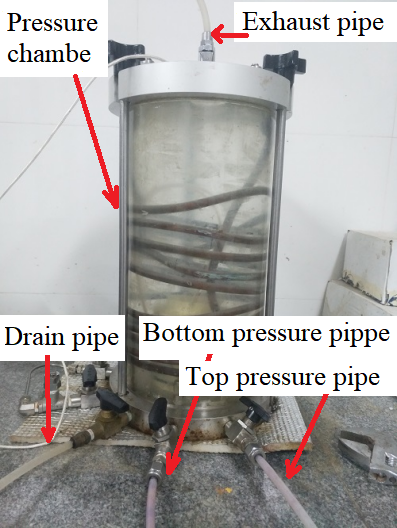 |
| (b) Loading specimen | (c) Specimen chamber |

**Supplementary Figure S3.** Description of permeability test instrument


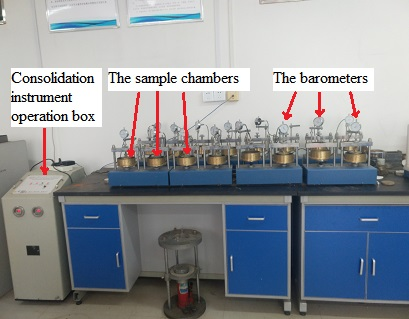


**Supplementary Figure S4.** Description of consolidation test instrument
